# Round the London Hospitals

**Published:** 1916-01-01

**Authors:** 


### ROUND THE LONDON HOSPITALS.

In most, indeed in practically all the London hospitals, Christmas Day was observed much in the usual way, and although the chastening influence of the war tended to keep the festive spirit within bounds, there was everywhere feasting and merrymaking. And the day which in some homes was the gloomiest of the year was in the hospitals the brightest, for the atmosphere of sadness that pervaded many a house that was full of happiness twelve months ago was not allowed to penetrate into the sick wards of children and wounded soldiers. The perplexities which disturbed the public generally in planning their Christmas holidays—whether they should be spent joyfully as befits the season, or soberly as befits the times—were not experienced by those whose place it is to order the doings of hospitals; and so every effort was strained to give the inmates of these institutions a happy day, and a very happy day it was.

A representative of THE HOSPITAL who visited most of the large London hospitals during the day brought away with him memories of merry Christmas parties at which plenty and good cheer were the presiding deities, of the singing of carols which heralded the dawn of Christmas even in the days when some of our oldest foundations were still young, and of many, many people poor in body but for the time being rich in the possession of a mind free from cares. All this is as it should be; for why should little children and the sick poor and wounded soldiers be deprived of a day's enjoyment, even though the whole world were in arms? It would hasten peace not by a single day if our hospitals contrived to forget Christmas—if Santa Claus failed the children, and if, instead of the turkey and the plum-pudding, the ordinary everyday rations were served out.

As one walks through the wards of a general hospital on Christmas morning one is impressed by the extraordinarily large proportion of patients who seem to be in really good health. Our representative remarked upon this curious fact to an official who was guiding him through one of the big institutions. "It is really wonderful," the official replied, "how few people, even in a hospital, seem to be really ill on Christmas Day." But, alas! it is the spirit rather than the body that is healed, and when the day's merriment is over the head again lies wearied on the pillow; the spirit was not healed—only stimulated; but all the better for the awakening.

### CHRISTMAS MORNING.

At all the hospitals the plans of entertainment were much the same—perhaps rather more lavish at some than at others—but the groundwork was very similar. At some there was more holly, at others more music; but everywhere a superabundance of turkey and plum-pudding—the basis on which the structure of the whole entertainment was based. At St. Mary's our representative arrived just as the steward was preparing to distribute gifts which a generous person had sent for each of the soldier patients, and was kindly allowed to be the steward's companion in this pleasant mission; and very grateful were the patients for these timely tokens. At this institution the decorations were both abundant and tasteful, being unsurpassed by those seen at any of the others visited. Lingering in the wards one could hear the strains of a Christmas hymn from the chapel, where the morning service was then proceeding; and very touching and beautiful did the music sound.

The visit to the Middlesex happened to time with the last basting of the turkeys, a process which our representative was permitted to witness; and as he waited in the kitchen he saw a long procession of more than twenty of these fine creatures pass through the door on the way to their several destinations. Before each dish left it was garnished with a liberal supply of sausages, served from a pan huge enough to furnish each of the many birds that had been cooked. He was shown the potatoes with their jackets on steaming in their nets—one net for each ward—and in fact all the secrets of this busy kitchen. At this hospital there are forty soldiers, and special efforts were made to give them a good time. The Christmas-tree was still fully loaded at the time of our representative's visit, but the children had already had a visit from Santa Claus, and even one or two dear little mites who were too poorly to take an interest in anything else were making friends here with a woolly doll and there with a box of bricks. As at some of the other hospitals, the arrangements for Christmas were rather less elaborate than usual, and one can well understand that with a shortage of doctors and nurses and a surplus of patients opportunities for preparation are not numerous; but there was carol singing by the nurses as usual, and if the day's entertainment was less varied than in normal times it was certainly none the less enjoyable.

LITTLE CHILDREN AND WOUNDED SOLDIERS.

As at all the other institutions visited, the most honoured guests at University College Hospital were the children and the soldiers. The ordinary patients were by no means forgotten, but special pains were taken to make the Christmas a memorable one for our sick and wounded heroes and a merry one for the children. There must be some hidden corner of the country in which turkeys are specially bred and fed for hospitals, for the Gargantuan birds one meets in the corridors are quite a few sizes larger than those one sees in the shops; they tell the writer that one of these turkeys will feed ten soldiers and fifteen ordinary patients, and if the calculation is a just one there is nothing amiss with the appetites of either class of patient. In the various military wards the soldiers were grouped round a central table, and a very happy party they made. Nothing of sadness was noticeable to the casual observer of this Christmas feast, and yet what tales of suffering these men could tell! The lusty cheers which welcomed the arrival of the colossal puddings suggested that in spite of wounds and in spite of tragic experiences these men are full of manly vigour. As one entered the children's ward one was greeted by the sound of cheerful little voices; some of the young folks were spending the interval between courses with their toys, too busy to take notice of anything else, while others of a more sociable turn were talking—not in whispers—to their friends a few cots away. One podgy little chap had left half his minced turkey and confessed to the writer that he would rather play with his toys than eat the rest.

DREAMING OF FATHER CHRISTMAS.

In the midst of all the Christmas Day festivities the ordinary work of a hospital must still go on. Thus, at the London our representative found several rows of out-patients waiting their turn to see the doctor. The motto most unhappily chosen by the trading community early in the war, "Business as usual," is just as applicable to a hospital on Christmas Day as at any other time. Here, if one doubted it, was evidence that people are taken ill even on Christmas Day, and before the round of festivities was begun there were operations to be performed. Dinner was over, the plates were cleared away, crackers had been pulled, and everybody seemed to be decked with paper caps. The house surgeons who had helped with the carving were still wearing their large cooks' hats and big white aprons, and everybody was in merry mood. The soldiers, some of whom seemed to have been bombarded with gifts, were particularly lively, and their presence certainly helped to cheer everybody else. In one of the children's wards visited the scene was more like a party at some rich man's house than a sick room. The little inmates were dressed in the fine raiment that does duty on these occasions, and the beating of drums, the weird sounds emitted by the weirdest of toys, and happy children's voices blended to make such discord as would sound like harmony even to a crotchety old man. The Christmas-tree—a wonderful affair standing in the centre of the ward—was still laden with toys, although the distribution was over and each child had received several gifts. A visitor might imagine that with such an embarrassment of choice as this tree had afforded the children would be torn by conflicting emotions in making their selection: should it be that highly coloured ball, or this long-eared animal without a tail? As a matter of fact there was no such difficulty. "You would have been surprised," said the sister, "to have seen how each one knew unhesitatingly which precise toy he wanted and meant to have." One or two had fallen asleep in the midst of the merriment, and one little fellow dressed in a blue suit was peacefully lying in his cot clutching a tiny trumpet all unconscious of what was going on around him, possibly in his dreams seeing Father Christmas emerging with sooty whiskers from the chimney. What a tragedy it would be if any of these children from impoverished homes should be well enough to leave the hospital on Christmas Eve!

A MUSICAL AFTERNOON.

On his arrival at St. Bartholomew's our representative was greeted by many musical sounds coming from all sides of the vast building. Standing in the centre of the quadrangle one could listen to the blending of all sorts of song: a Tipperary chorus from the East, a pathetic solo from the West, and a glee from the South. The whole place seemed to exude pleasant sounds, but as it is always preferable to hear the various classes of music separately, he made a tour of the wards in which these strains were produced. Here concert parties were in full swing. A pierrot troupe, composed mainly of pierrettes, was giving a most excellent performance, which was obviously much appreciated by the audience. Another party, known as "The Dry Dressings—Resterilised," into whose identity we will not examine too closely, had a well-chosen repertoire of the kind of songs which everyone can appreciate on Christmas Day; they put their heart into the business, and were well repaid by the applause of the patients. There were other troupes and other performers giving great pleasure wherever they went.

At St. George's Hospital plenty of entertainment was provided. Visitors were received by the patients, everything was done to make the wounded soldiers happy, and the children had a Christmas-tree. In the afternoon there was a concert, and altogether a most enjoyable day was spent.

At Charing Cross Hospital, which contains a large number of wounded, Christmas Day was a great festival. Elaborate entertainments were organised by Mr. Athol Stewart. The programme on each floor was under the direction of some notable actor or stage manager, each of whom was assisted by a number of artistes who gave their services.

The festivities at the Chelsea Hospital for Women were on a more limited scale than usual. On Christmas Day itself the Ladies' Committee arranged for dinners, and other customary celebrations took place whilst the patients had the opportunity of singing Christmas hymns and of entertaining their friends at tea.

The general impression gained from the tour was that the merriest and happiest place to be in on a Christmas Day is a voluntary hospital.

